# The FloRes Database: A floral resources trait database for pollinator habitat-assessment generated by a multistep workflow

**DOI:** 10.3897/BDJ.10.e83523

**Published:** 2022-09-14

**Authors:** Franziska Baden-Böhm, Mario App, Jan Thiele

**Affiliations:** 1 Thünen Institute of Biodiversity, Braunschweig, Germany Thünen Institute of Biodiversity Braunschweig Germany; 2 Institute of Geoecology, Technische Universität Braunschweig, Braunschweig, Germany Institute of Geoecology, Technische Universität Braunschweig Braunschweig Germany

**Keywords:** pollinators, bumblebees, hoverflies, floral resource, pollen, nectar, corolla, phenology, sugar concentration, protein, habitat assessment

## Abstract

**Background:**

The decline of pollinating insects in agricultural landscapes proceeds due to intensive land use and the associated loss of habitat and food sources. The feeding of those insects depends on the spatial and temporal distribution of nectar and pollen as food resource. Hence, to protect insect biodiversity, a spatio-temporal assessment of food quantity of their habitats is necessary. Therefore, sufficient data on traits of floral resources are required.

**New information:**

As floral resources’ traits of plants are important to quantify food availability, we present two databases, the FloRes Database (Floral Resources Database) and the raw database, from where FloRes was derived. Both databases contain the plant traits: (1) flowering period, (2) floral-unit density per day, (3) nectar volume per floral unit per day, (4) sugar content per floral unit, (5) sugar concentration in nectar, (6) pollen mass or volume per floral unit and per day, (7) protein content of pollen and (8) corolla depth. All traits were sampled from literature and online databases. The raw database consists of 702 specified plant species, 138 unspecified species 37 species (spec., sp), 22 species *pluralis* (spp) and, for 79, only the genus was identified) and two species complexes (agg.). Those 842 taxa belong to 488 genera and 102 families. Finally, only 27 taxa have a complete set of traits, too few for a sufficient assessment of spatio-temporal availability of floral food-resources.

As information on floral resources is scattered throughout many publications with different units, we also present our multistep workflow implemented in five consecutive R-scripts. The multistep workflow standardises the trait units of the raw database to comparable entities with identical units and aggregates them on a reasonable taxonomic level into the second application database, the FloRes Database. Finally, the FloRes Database contains aggregated information of traits for 42 taxa and, when corolla depth is excluded, for 72 taxa.

This is the first attempt to gather these eight traits from different literature sources into one database with a multistep workflow. The publication of the multistep workflow enables the users to extend the FloRes Database on their own demands with other literature data or newly-gathered data to improve quantification of food resources. Especially, the combination of pollen, nectar and the open flowers per square metre is, as far as we know, a novelty.

The FloRes Database can be used to evaluate the quantity of food-resource habitats available for pollinators, for example, to compare seed mixtures of agri-environmental measures, such as flower strips, considering flower phenology on a daily basis.

## Introduction

The intensive management of land, the associated loss of feeding, shelter and nesting habitats (Tilman et al. 2002, Carvell et al. 2007, Beckmann et al. 2019) and the resulting lack of floral resources in natural and agricultural landscapes in space and time have affected pollinators' diversity and abundance (*Brown and Paxton 2009, Potts et al. 2010, Williams et al. 2012, Cardoso et al. 2020*). Restoring and establishing semi-natural habitats and agri-environmental measures, for example, hedgerows, meadows or flower strips, can mitigate the decline of pollinating insects through increasing the supply of floral resources (Korpela et al. 2013, Pywell et al. 2015, Carvell et al. 2017).

Pollinators, such as bees and hoverflies, rely on nectar as an energy source for movement and vital processes, as well as on pollen for reproduction (*Haslett 1989, Potts et al. 2003, Westrich 2018*). The availability of pollen and nectar must be ensured throughout the season, without temporal gaps in resource availability in order to prevent a decrease of pollinator populations (Roulston and Goodell 2011). Therefore, quantification of the spatio-temporal distribution of floral resources is crucial for assessing the potential of habitats and landscape sections to support pollinators. Many researchers pursue the assessment of habitats for pollinating insects, which require knowledge about quantity, quality and phenology of floral resources, i.e. nectar and pollen (*Potts et al. 2003*). Especially phenology and corolla depth have an important impact on the form of the ecological niche of pollinators (Junker et al. 2013) and, thus, should be considered for species-specific habitat assessment.

Consequently, the spatio-temporal quantification of nectar and pollen supply for pollinators demands knowledge of:

1. Physiological traits of the flower phenology and flower density
2. The quantitative amounts of the floral resources per flower
3. The availability of nectar to pollinators determined by the corolla depth.

Thus, information of phenology, floral unit density, nectar volumes or sugar amount of floral unit, sugar concentration, pollen per floral unit, protein content of pollen and corolla depth, based on literature and existing databases of plant traits, is required.

Already existing data (e.g. Baude et al. 2016, Hicks et al. 2016, Ouvrard et al. 2018) cover different aspects of the required traits. Baude et al. (2016) published a comprehensive dataset for sugar per day and flower, as well as the density of open flowers [m^-2^], but the values for pollen were lacking. The data of Hicks et al. (2016) and Ouvrard et al. (2018) also included data of pollen per day and flowers, but the density of all open single flowers per area was lacking. Few datasets, like Becher et al. (2018) for the agent-based model BumbleBEEHAVE, covered all those traits, but the variety of species was too small for improved assessment of temporal quantification of nectar and pollen supply for pollinators.

Hence, we compiled a raw database composed of the demanded floral resource traits, based on literature and existing databases of plant traits, to increase the amount of plant taxa. Data on phenology and abundance are comparatively easy to acquire and, therefore, well available, but data on floral resources and corolla depth are scattered. As the raw database contains very few species with a complete set of traits and often traits do not refer to same level of the inflorescence (e.g. single flower or capitula, umbel etc.), we generated a second application database, the FloRes Database through a multistep workflow. This multistep workflow sets nectar and pollen in relation to the floral unit and then aggregates them on a reasonable taxonomic level. A floral unit is defined from the perspective of the insects as the number of flowers that can be visited without flying, ranging from a single flower up to thousands (Carvalheiro et al. 2008). FloRes contains much fewer species than the raw database, but includes more taxa with a complete set of traits.

Thus, with FloRes Database, we start filling a knowledge gap about the floral resources provided to pollinators and their spatio-temporal provisioning to pollinators. Our database enables us to quantify nectar and pollen per habitat area and day throughout the seasons, allowing us to find temporal provisioning gaps. Due to the spatial connection of pollen and nectar with floral units per area, we are also able to estimate the floral resources in whole habitats and landscapes. The floral units per area can be easily divided through the specific habitat cover percentage of a plant to achieve this. In this way, food sources in habitats, such as semi-natural habitats and agri-environment measures, can better be described and assessed for pollinators. Such data can also be useful to compare flowering habitats or seed mixtures (Hicks et al. 2016). If the corolla depth of plant species and the proboscis length of a pollinator are known, species-specific access to nectar can be calculated. To enable other users, as well to add data on flower traits, we published also our workflow written in R (R Core Team 2021).

## Sampling methods

### Sampling description

We collected data for eight floral traits (Table 1) from 34 published articles and their supplementary materials as well as from two books, reports and dissertations each and from an online database (“References_of_raw_data.pdf”, Dryad repository in folder attachment). For cultivar plants, the traits were sampled mainly in field experiments. For wild flowers, the entities were recorded either on natural, semi-natural habitats or botanical gardens. Most research was done in Europe, especially Poland and England, though some information came from a Northern America database (see “Geographic_Information_raw_data.pdf”, Dryad repository in folder attachment).

For the raw database, some input data of the traits had to be adapted. We calculated the average molar sugar concentration per species from the data of *Gilbert (1981)* assuming the sugar is pure saccharose. If the values of corolla depth were 0 in the reference of *Gilbert (1981)* and Becher et al. (2018), the species have open flowers. For species in *Becher et al. (2018)* which only provide pollen, but no nectar, we set corolla depths to NA. If the nectar volume was 0 in Becher et al. (2018), but other references recorded a nectar volume or nectar sugar content > 0, the values were not transferred into the database.

For the quantitative traits, we gathered minimum, maximum and mean values, if available. With the traits 'pollen', 'nectar volume', 'sugar per flower' and 'flower' or 'inflorescence density', we recorded the flower unit they referred to, i.e. either per single flower or per inflorescence. The reference flower unit is very important for scaling nectar volume, nectar sugar content and pollen to the same flower unit, enabling merging and aggregation of trait data from different sources. Furthermore, the nomenclature of species varied in literature. Therefore, we equalised the species names in our database in column 'species' in our database, but we also included the names used in the original publications to facilitate joins and backtrackings with the data source (column 'species_name_reference' in our database).

**Data preparation and multistep workflow**

We compiled the FloRes Database so as to include as many species/taxa with a complete set of traits as possible through a multistep workflow in R 4.1.0 (R Core Team 2021) using five consecutive scripts:

1. We converted flowers and inflorescences per square metre as well nectar and pollen per flower or inflorescence to the level of floral units using Formulae 1 and 2 (Script: 1_Inflorescences.R). This step requires the dataset "AgriLand_FlowerDensity_perspecies.csv" of *Baude et al. (2015)*.
2. We converted trait values to the same physical units for each trait and calculated missing trait values from other traits using Equations 3 to 5 and 7 to 9 (Script: 2_units.R).
3. We took the means (except for flowers per square metre where we used the maximum) of multiple trait entries for each species (Script: 3_Aggregate_species.R).
4. We either unified synonymous species names or grouped species on a reasonable taxonomic level (taxon) for the next step to combine and aggregate the plant species. Further, we deleted those with few entries (Script: 4_Selecting_taxa.R). The grouping of species is given in the required auxiliary file “Taxa_to_aggregate.csv”, which can be edited.
5. We calculated the means of the traits of the synonymous species and repeated, now with the more complete dataset, the derivation of traits from other traits using Equations 4 and 6 (Script: 5_Aggregation_selected_taxa.R).

The first and second script were used to convert the data to equal units, whereas scripts three to five were used to aggregate and combine the trait data on the most suitable taxonomic level, preferably on species level (Column 'taxon' in our database). However, we could frequently aggregate only on genus level.

**Conversions of units**

Throughout the literature, flowers were defined in different units and were given in different units. Therefore, the flower units needed to be transformed to the same definition of floral units (Carvalheiro et al. 2008) per area. This enabled us to scale pollen and nectar availabilty correctly per area.

- **Floral units per area**

For assessing the quantity of floral resources on habitat scale, the quantity of pollen or nectar (sugar) per standardised area is needed. In the raw data, the floral reference unit, either single flower or inflorescence, sometimes varied between flower-unit density, nectar and pollen data. For simplification the terms raceme, panicle, corymb, globular raceme, umbel or catkin in the database of Baude et al. (2015) were defined by us as inflorescences for the reference floral unit. Therefore, in order to facilitate calculations of nectar or pollen per square metre, we used the entity of the floral units (Carvalheiro et al. 2008) given in *Baude et al. (2015)*. Thus, single flowers could be summed up to inflorescences or inflorescences down to single flowers. Therefore, we transformed abundances of single flowers *f_a_* [m^-2^] into abundances of floral units *fu_A_* [m^-2^] through division by the number of open flowers per inflorescence *f_I_* using the information from Baude et al. (2015) (Equation 1).

\begin{varwidth}{50in}
        \begin{equation*}
            fu_A=f_A/f_I 
        \end{equation*}
    \end{varwidth} (1)

- **Pollen and nectar per floral unit**

Floral resources *R_f_*, i.e. pollen or nectar, were multiplied with the number of open flowers per floral unit *f_i_* (*Baude et al. 2015)* to obtain the floral resource *R_fu_* per floral unit:

\begin{varwidth}{50in}
        \begin{equation*}
            R_{fu}=P_f⋅f_i
        \end{equation*}
    \end{varwidth} (2)

For open flowers per inflorescence of *Helianthusannuus*, we used data from Minckley et al. (1994), because *H.annuus* was not recorded in *Baude et al. (2015)*.

For floral units per area, we used the maximum value, not the mean, when there were multiple values per species. Here, we used the maximum density as an approximation for 100% cover of the plant species. This allowed us to scale the floral resource per square metre in a given habitat, when the habitat specific cover percentages of plant species were available. The floral unit density per area needed to be divided through the cover percentage.

- **Nectar volume, nectar sugar content and sugar concentration of nectar**

Mostly, nectar was measured as secretion of liquid per flower and day [volume flower^-1^ d^-1^] (e.g. *Bosch et al. 1997, Hedtke 2000, Horn 2017, Becher et al. 2018*) or as the sugar content per flower and day [mass flower^-1^ d^-1^] (e.g. *Crane et al. 1984, Maurizio and Schaper 1994, Baude et al. 2016, Hicks et al. 2016*). We followed this convention and calculated both the volume [ml floral unit ^-1^ d^-1^] and sugar content per flower [mg floral unit^-1^ d^-1^]. For the conversion of nectar volume to sugar content and vice versa, we used Equations 3 to 6.

To receive sugar in mass per flower *m_z_* [mg], we multiplied nectar volume *V_nec_* [ml] by density of saccharose *ρ_z_* [1570 mg ml^-1^] and sugar concentration *c_perc_* [%]:

\begin{varwidth}{50in}
        \begin{equation*}
            m_z = \rho_z V_{nec} \frac{c_{perc}}{100}
        \end{equation*}
    \end{varwidth} (3)

When only molar concentration of sugar *c_mol_* [mol l^-1^] was given, we used the following equation with molar mass of saccharose *M_z_* [342.3 mg mol^-1^]:

\begin{varwidth}{50in}
        \begin{equation*}
            m_z = V_{nec} M_z \frac{c_{mol}}{1000}
        \end{equation*}
    \end{varwidth} (4)

To calculate the nectar volume *V_nec_* [ml] from sugar content per floral unit *m_z_* [mg], we used:

\begin{varwidth}{50in}
        \begin{equation*}
            V_{nec} = \frac{m_z} {\rho_z⋅c_{perc}} 100
        \end{equation*}
    \end{varwidth} (5)

When *c_perc_* was not given, we used *c_mol_* to calculate *V_nec_*:

\begin{varwidth}{50in}
        \begin{equation*}
            V_{nec} = \frac{m_z} {M_z c_{mol}} 1000
        \end{equation*}
    \end{varwidth} (6)

For our application, we needed the sugar concentration in molar concentration, so we transformed *c*_perc_ to molar concentration as:

\begin{varwidth}{50in}
        \begin{equation*}
            c_{mol} = c_{perc} \frac{\rho_z} {M_z} 10
        \end{equation*}
    \end{varwidth} (7)

- **Mass of pollen per floral unit and pollen protein content**

For pollen, the physical units differed due to extraction methods. Mostly, the mass of pollen was given and the values only needed to be scaled to mg, if given in g or µg. However, sometimes it was given as estimated volume of pollen grains (Hicks et al. 2016). Therefore, we used:

\begin{varwidth}{50in}
        \begin{equation*}
            m_p=V_p * \rho_p
        \end{equation*}
    \end{varwidth} (8)

to calculate pollen mass *m_p_* [mg] from the pollen volume *V_p_* [ml] and the pollen density *ρ_p_* [mg ml^-1^]. Since for most species, the density of fresh pollen *ρ_p_* is not known, we used:

\begin{varwidth}{50in}
        \begin{equation*}
            \rho_p = \rho_{prot} P_{prot} + \frac{(1 - P_{prot})}{3} (\rho_{starch} + \rho_{fat} + \rho_{water})
        \end{equation*}
    \end{varwidth} (9)

with mean densities of protein *ρ_prot_* [1300 mg ml^-1^] (Chick and Martin 1913), of starch *ρ_starch_* [1440 mg ml^-1^] (Marousis and Saravacos 1990), fat *ρ_fat_* [900 mg ml²] (*Deutsches Institut für Normung e.V. 2002*) and water *ρ_water_* [1000 mg ml^-1^] and with the proportion of protein of the pollen *P_prot_*[-]. When *P_prot_* was not given for a plant species, we estimated it through mean protein content of the genus, as the protein contents are relatively similar amongst the species of a genus (*Roulston et al. 2000*). When the protein amount could not be estimated for a genus, it was estimated as the mean of all species in the database.

**Aggregation of data, replacement of synonyms and FloRes Database**

After equalising floral, physical and chemical units (script: 2_units.R), we aggregated the traits using the mean of multiple entries per taxon, except for the case of floral units per area, where we used the maximum value to receive an approximate density of floral units at 100% coverage of the species (script: 3_Aggregate_species.R). Subsequently, we checked the species for completeness of traits and grouped closely-related taxa with incomplete, but complementary, trait information in a table (Taxa_to_aggregate.csv) for further aggregation on genus level or a reasonable higher taxonomic level. We used this table to add how plant species should be automatically aggregated in the script 4_Selecting_taxa.R. Moreover, we used this step to aggregate the synonymous names of a species with their common name or on a higher taxonomic level. Finally, we aggregated the traits a second time by the selected taxon (species, genus or higher level). In cases where values for molar sugar concentration were still lacking after Step 5, we inserted the average value of 40% of sugar concentration as an estimate for wildflowers, as given in Westrich (2018).

After the final aggregation, we got three different output tables for the FloRes Database. “5_FloRes_raw” contains the mean values of all taxa for which at least some trait data were available. “5_FloRes_complete_trait” is the dataset of taxa without any gaps. “5_Selected_taxa_no_corolla” contains taxa where all traits, except for corolla depths, were complete. Those datasets can finally be used to calculate the amount of nectar and pollen of habitats within any defined time period, given that the plant species of the habitats are included in the database.

## Geographic coverage

### Description

The database is a collection of data from the Northern Hemisphere, focused on Central Europe. The details about the geographical information of the raw database references are listed in https://datadryad.org/stash/share/pYjuf_kRaA0N9Lw25svZa_rnQ_mENIIyQAC2rkXicEI.

## Usage licence

### Usage licence

Creative Commons Public Domain Waiver (CC-Zero)

## Data resources

### Data package title

The FloRes Database: A floral resources trait database for pollinator habitat-assessment generated by a multistep workflow

### Resource link

https://doi.org/10.5061/dryad.djh9w0w29

### Number of data sets

2

### Data set 1.

#### Data set name

Data.zip

#### Data format

.csv

#### Download URL

https://doi.org/10.5061/dryad.djh9w0w29

#### Description

The raw data, all intermediate and auxillary datasets and the final FloRes Database are published in the Dryad repository.

In the raw database, the same traits are covered, but the units and the dependent flower units are given in extra columns ending on the ”*_unit*” and “*_regarding_flowering_unit*”. Further, the literature citation is given in the column ending with “*_references*”.

**Data set 1.** 

Column label	Column description
Phenology	Flower life span and flowering start and end given as day of the year [d].
Flower unit density	The number of single flowers or inflorescences per square metre [m²].
Nectar volume	The nectar volume per single flower or inflorescences [ml].
Sugar concentration in nectar	The concentration of sugar in nectar in mol per litre [mol l^-1^] or percentage [%]. Sugar is assumed to be pure saccharose.
Sugar per flower	The absolute sugar content of nectar per single flower or inflorescences [mg].
Pollen	The pollen per single flower or inflorescences [mg or ml].
Protein content of pollen	The amount of protein in pollen [%].
Corolla depth	The depth of the corolla tube [mm].

### Data set 2.

#### Data set name

multistep_workflow_scripts.zip

#### Data format

.R

#### Download URL

https://doi.org/10.5061/dryad.djh9w0w29

#### Data format version

R.-4.1.0

#### Description

All scripts used for generating the FloRes Database from the raw data.

**Data set 2.** 

Column label	Column description
none	none

## Additional information

### Data Statistics

**Raw database**

The raw database consists of 702 specified plant species, 138 unspecified species (37 species (spec., sp), 22 species pluralis (spp) and, for 79, only the genus was identified) and two species complexes (agg.). Synonyms of species names are not counted as the same species. All 843 taxa belong to 448 genera and 102 families.

**Figure 1.**
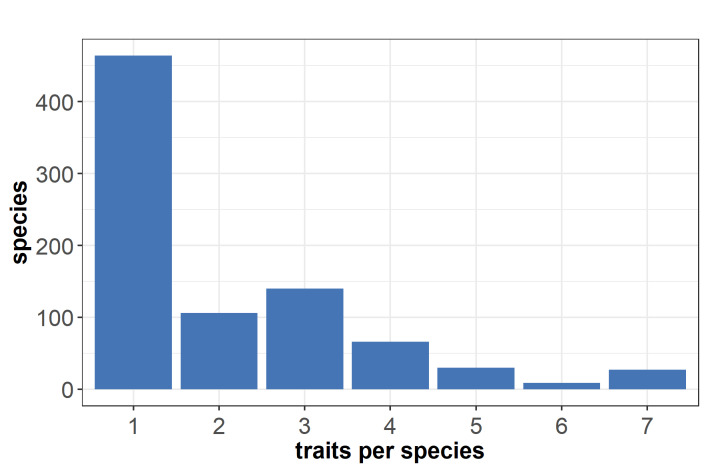
Number of species in the raw database which had at least one entry per trait. The traits comprised phenology, floral unit density per area, corolla depth, nectar volume per floral unit and day, sugar concentration in nectar, pollen per floral unit and day, as well as protein content of pollen.

**Figure 2.**
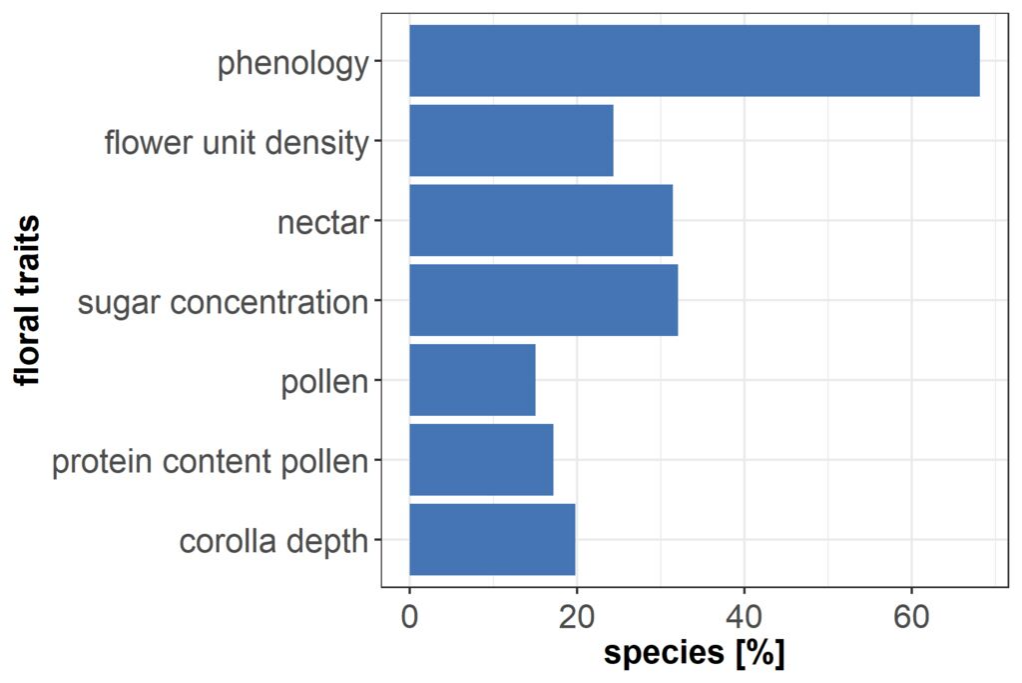
Percentage of species with minimum one or more entries per trait in the raw database.

Most of the collected species had either data for one or few traits (Fig. 1). Only few species had entries of four to seven traits of interest. Only 27 had a complete set of traits of interest. To explore the quality of the raw database, the percentages of the species with one or more entries per trait were plotted (Fig. 2). Flowering period had the fewest species with lacking entries. Yet, less than 40% of species were provided with data for each of the other traits.

Hence, most of the species were insufficiently provided with trait data. Therefore, it was necessary to combine and aggregate species on a reasonable taxonomic level for a comprehensive habitat assessment.

**FloRes Database**

After aggregating and combining the traits of the same species or closely-related taxa, 42 taxa with a complete set of traits remained in the FloRes Database. Those taxa belonged to 38 genera and 17 families. When excluding corolla depth, the numbers increased to 72 taxa from 63 genera and 22 families.

**Figure 3.**
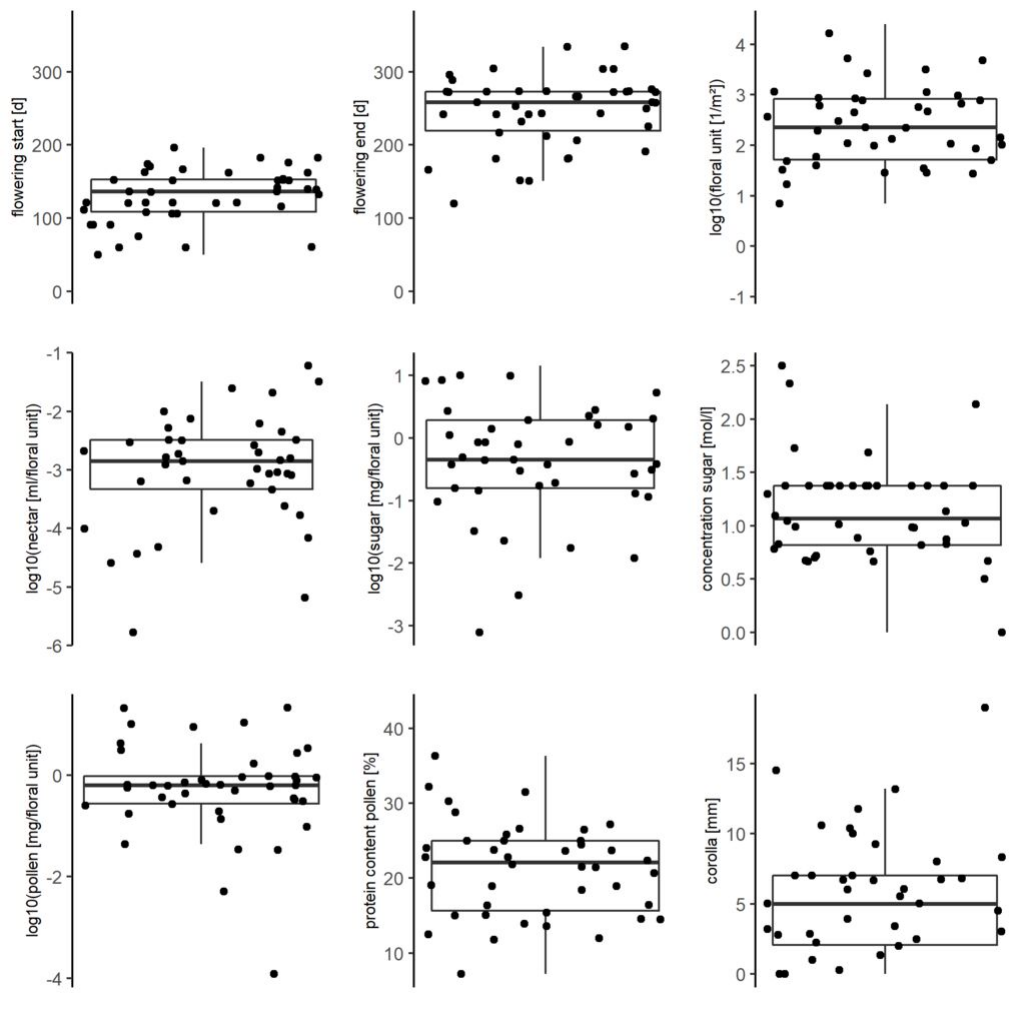
The distribution of magnitude of each floral trait which is contained in FloRes Database (Floral Resources Database) using only the 42 plant taxa with entries in all traits. The lower/upper boundary of the boxes shows the 25%/75% quantile and the line dividing the box represents the median. All original values per floral traits are depicted as point scattering over the boxplot.

**Figure 4.**
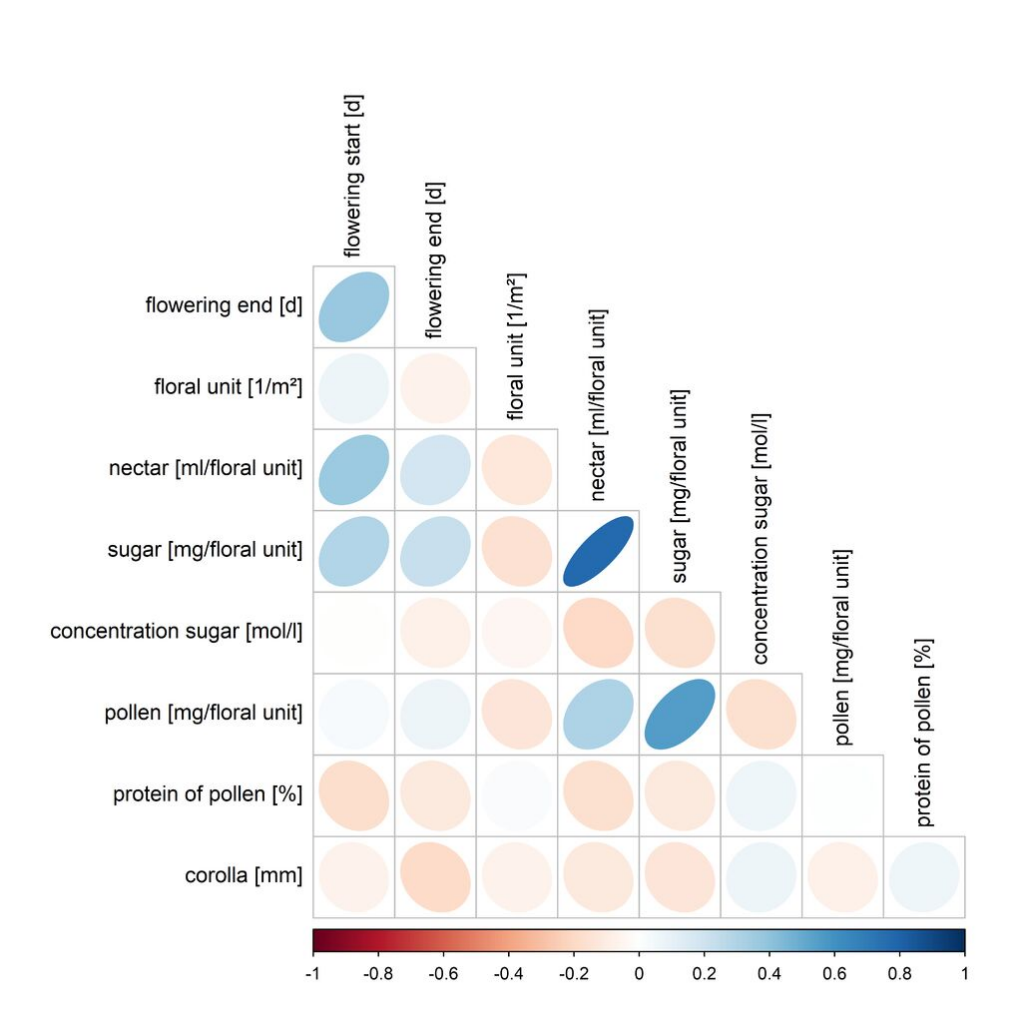
Pearson coefficient correlations between the species’ traits. Red colour scale (-1 to 0) indicates negative linear correlation and blue colour scale (0 to 1) positive linear correlation between two traits. White indicates no linear correlation (0) between two traits. The stronger the relationship, the darker the colour and the circle shape becomes more and more elliptical.

All traits varied strongly amongst the taxa (*Fig. 3*). Most remarkable was the huge span of the floral units per square metre and of nectar and pollen per floral unit, ranging across three to five orders of magnitude. Correlations amongst traits were mostly weak or moderate (Fig. 4). However, there was a strong positive correlation (r = 0.78) between nectar volume and sugar per floral unit (t = 7.8575, df = 40, p-value = 1.234e-09), which is in line with the moderate variation in sugar concentration (*Fig. 3*). Further, there was a positive correlation (r = 0.56) between pollen and sugar per floral units (t = 4.2479, df = 40, p-value = 0.0001251), which could be explained by larger floral units spending more sugar and pollen. Correlations were calculated with Pearson's correlation coefficient using R (R Core Team 2021). Significance levels of correlations were also calculated with R.

### Limitations and uncertainties

We did not collect our own data in the field or laboratory, but we gathered trait values from different sources. Thus, we often did not know if the density of the floral units referred to 100% cover of the plant species in its habitat. When not specified, we assumed the highest given density as 100% cover, which is only a rough estimation. Additionally, it was unknown in which habitats the flowers per area were counted. Therefore, an accurate estimation of nectar and pollen supply on habitat levels is hampered. In addition, the volume of nectar per flower varies per day and also within the day. The diurnal rhythm was not considered. Further, the sugar content in nectar depends on the soil moisture and air humidity (Westrich 2018). Additionally, the nectar volume and sugar content per floral unit were derived from mass, where necessary, assuming molar mass and density of saccharose, although nectar is often a mixture of glucose, fructose and saccharose (*Percival 1961*).

Frequently, pollen is given in grains or volume and without exact measurements of pollen densities. Therefore, the values of pollen mass derived from volume are rough estimates, because the fat-carbohydrate-protein composition of pollen is mostly unknown. As well, there was very little information about anther position, which may limit the physiological accessibility of the pollen (Junker et al. 2013), so there is no species-specific estimation of pollen availability possible. The form of the ecological niche is further influenced by flower heights and floral reflectance (Junker et al. 2013), which FloRes does not consider, because we have found little information about them.

Hence, in its current state, the FloRes Database can provide a rough estimation on quantity of species-specific floral food resources.

### Applications

Potential application of the database is the description and evaluation of the quantity of available food resources plant species provide on a habitat scale.

This allows us evaluate the temporally available floral resources in a given time period of, for example, days, weeks or months of existing seed mixtures for flower strips or other agri-environmental measures as similary is done in Hicks et al. (2016). Alternatively, new ones can be created, which ensures temporal continuity of available floral resources throughout the year.

In our own research, we applied the FloRes Database to generate input data of nectar and pollen supply of habitats for spatial and temporal explicit simulation models of bumblebee and hoverfly populations, to evaluate the effects of landscape composition and configuration on both species. For bumblebees, we used the agent-based model (ABM) BumbleBEEHAVE with the model BEESCOUT_2.0 (*Becher et al. 2018*) and, for aphidophagous hoverflies, we developed the yet-unpublished SyrFitSources. Both models simplify raster data of habitats types into spatial points, called by us patch-agents and carry the information of area and floral unit densities (for the algorithm, see Becher et al. 2016). Additionally, SyrFitSources connects the patch-agents with a habitat network, based on euclidean distance. In both models, the daily amount of nectar and pollen per habitat is calculated as dependent on habitat-specific plant taxa coverage to estimate the daily available resources either for bumblebees or hoverflies. Therefore, we used the FloRes dataset "5_FloRes_no_corolla". However, before we could apply the data as a base for model input of nectar and pollen amount, we had to fill in missing values of the corolla depths through educated guesses for each target species, enlarging the number of usable taxa of plants from 42 up to 70. For our specific models, the units of the traits needed to be converted or column names had to be changed.

Finally, the described workflow and the published scripts allow us and other users to easily expand and improve the FloRes Database by simply adding new lines to the raw database. This will facilitate a steady increase of bundled information of floral resources to improve the assessments of spatio-temporal food availability in habitats for pollinators.

## Figures and Tables

**Table 1.** Traits in raw and FloRes database

**Trait**	**Coded in database**	**Description**	**Units raw database**	**Units FloRes database**
**Physiological traits**				
Phenology	flowering flowering_start flowering_end	Flower life span and flowering start and end given as day of the year	d	d
Flower unit density	flowers_m2	The number of open single flowers or inflorescences per square metre [m²]	single flowers/m² or inflorescences/m²	floral units /m²
**Floral resources**				
Nectar volume	nectar_volume	The nectar volume per single flower or inflorescences	µl/d, ml/d, l/(m²d)	ml/d
Sugar concentration in nectar	sugar_conc	The concentration of sugar in nectar in mol per litre and percentage	mol l^-1^, %	mol l^-1^, %
Sugar per flower	nectar_sugar_cont	The absolute sugar content of nectar per single flower or inflorescences	µg/d, mg/d	mg/d
Pollen quantity	pollen	The pollen per single flower or inflorescences	µg, mg, g, g/m², µl,	mg
Protein content of pollen	protein	The amount of protein in pollen	%, g/100g dry mass	%
**Resource availability**				
Corolla depth	corolla	The depth of the corolla tube	mm	mm
